# Urinary miRNA profiles discriminate between obstruction-induced bladder dysfunction and healthy controls

**DOI:** 10.1038/s41598-021-89535-3

**Published:** 2021-05-13

**Authors:** Michelle von Siebenthal, Mustafa Besic, Ali Hashemi Gheinani, Akshay Akshay, Salomé Lizun-Platoni, Nadine Kunz, Fiona C. Burkhard, Katia Monastyrskaya

**Affiliations:** 1 Urology Research Laboratory, Department for BioMedical Research DBMR, University of Bern, Bern, Switzerland; 2 Urological Diseases Research Center, Boston Children’s Hospital, Harvard Medical School, Boston, USA; 3 Broad Institute of MIT and Harvard, Cambridge, MA USA; 4 Department of Urology, Inselspital University Hospital, 3010 Bern, Switzerland

**Keywords:** Biological techniques, Molecular biology, Biomarkers, Medical research, Urology

## Abstract

Urgency, frequency and incomplete emptying are the troublesome symptoms often shared between benign prostatic obstruction-induced (BLUTD) and neurogenic (NLUTD) lower urinary tract dysfunction. Previously, using bladder biopsies, we suggested a panel of miRNA biomarkers for different functional phenotypes of the bladder. Urine is a good source of circulating miRNAs, but sex- and age-matched controls are important for urinary metabolite comparison. In two groups of healthy subjects (average age 32 and 57 years old, respectively) the total protein and RNA content was very similar between age groups, but the number of secreted extracellular vesicles (uEVs) and expression of several miRNAs were higher in the young healthy male volunteers. Timing of urine collection was not important for these parameters. We also evaluated the suitability of urinary miRNAs for non-invasive diagnosis of bladder outlet obstruction (BOO). A three urinary miRNA signature (miR-10a-5p, miR-301b-3p and miR-363-3p) could discriminate between controls and patients with LUTD (BLUTD and NLUTD). This panel of representative miRNAs can be further explored to develop a non-invasive diagnostic test for BOO. The age-related discrepancy in the urinary miRNA content observed in this study points to the importance of selecting appropriate, age-matched controls.

## Introduction

Patients with lower urinary tract dysfunction (LUTD) suffer a range of symptoms such as increased daytime and nighttime frequency, urgency, urinary incontinence, slow stream, hesitancy and incomplete emptying^1^. Continuous need for a bathroom, sleep cycle disruption, debilitating incontinence and frequent urinary tract infections strongly affect their quality of life and physical and emotional well-being^2^. An estimated 45% of the worldwide population is affected by at least one lower urinary tract (LUT) symptom^3^, which can be caused by various pathologies including bladder outlet obstruction (BOO), bladder pain syndrome, overactive bladder syndrome and neurologic diseases including spinal cord injury, Parkinson’s disease and Multiple Sclerosis. LUTD causes significant socioeconomic burden: in the USA, the healthcare costs in patients with an overactive bladder (OAB) were more than 2.5 times those of similar patients without overactivity^4^. It is one of the main reasons why the elderly are moved to a nursing home, further increasing the healthcare expenditure and hampering their life standard^5^. The incidence of multifactorial LUTD is growing concomitant with the ageing population.

BOO is characterized by an increased outlet resistance leading to an elevated detrusor pressure and decreased urinary flow during voiding. Urodynamic examination remains the gold standard procedure for the diagnosis of obstruction-induced LUTD^6^, but it is an invasive procedure, associated with risks of haematuria and urinary tract infection. It is expensive and needs specific equipment and expertise. Therefore, effort is being made to find non-invasive parameters for the diagnosis of BOO, avoiding the burden and morbidity associated with invasive urodynamics^7^. Urine is easy to collect, making it an attractive source of potential biomarkers. It contains small molecule metabolites and a considerable number of proteins and nucleic acids, both free and packaged in urinary extracellular vesicles (uEVs)^8^. Evidence of alterations in constituent urinary proteins in response to BOO comes from the early animal studies of partial BOO (pBOO)^9^, which identified a significant down-regulation of prostatic steroid-binding protein C3 precursor, glandular kallikrein 9 (S3) precursor, and glandular kallikrein 8 (P1) precursor. As research continued, a number of molecules were proposed as OAB or obstruction biomarkers, including urinary TGF-β^10^, cytokines and PGE2^11^, neurotrophins like nerve growth factor (NGF)^12^ and brain-derived neurotrophic factor (BDNF)^13^, and ATP^14^.

MicroRNAs (miRNAs), small non-coding single-stranded RNAs, are important modulators of gene expression^15,16^ and play a role in bladder pathologies^17–21^ and function^22^, including urothelial permeability^23^ and bladder contractility^18,24^. Urinary miRNAs have been proposed as biomarkers for diagnostic applications in urological malignancies^25^, but to date there is no information about the alterations of miRNAs in the urine of patients with LUTD. However, using human patients’ biopsies we have recently shown that a three-miRNA biomarker signature (miR-103a-3p, miR-10a-5p, miR-199a-3p) can discriminate between urodynamically-defined phenotypes of BOO-induced lower urinary tract dysfunction^26^. In order to address the question of urinary biomarkers for LUTD, we first established protocols for miRNA isolation from urine. Circulating RNAs can be packaged in secreted urinary extracellular vesicles (uEVs) and thus protected from degradation. Urinary exosome preparations, corresponding to uEVs with sizes of 50–150 nm, might contain specific miRNAs, which could be relevant as biomarkers in renal and bladder diseases. In our previous study^27^, we used five different methods to isolate uEVs and compared the size distribution, morphology, yield, presence of exosomal protein markers and RNA content of uEVs. We profiled the miRNA content of uEVs and total urine from the same samples with the NanoString platform and validated the data using RT-qPCR^27^.

Here we conducted a comparative study of the contents in urine, including uEVs and miRNAs in two groups of control subjects without LUTD (average age 32 and 57 year old, respectively). We analyzed control samples collected at 2 different time points during the day, because although samples from the first voiding of the morning are most suitable for biochemical testing^28,29^, the second voiding is more practical in the outpatient clinic^29^. Having established an appropriate sample collection protocol, we then compared the urinary miRNA profiles of the older control group with the samples from patients with benign prostatic obstruction-induced (BLUTD) and neurogenic (NLUTD) bladder dysfunction, and revealed significant changes in the miRNA content. Bladder function is affected by multiple diseases, with different triggering mechanisms and many confounding factors. A non-invasive urinary test discriminating patients with BLUTD from non-obstructed controls or patients with LUTD of different origin (such as neurogenic bladder dysfunction) can help the clinical symptom-based assessment and provide a less invasive approach than urodynamics, which would be of great benefit for patients. We propose a small panel of selected miRNAs, that can discriminate between patients with LUTD and controls. These selected miRNAs may upon further exploration have the potential for non-invasive urinary miRNA-based diagnosis of obstruction-induced bladder dysfunction.

## Results

### Study design

This study pursued two goals:

- assess whether the age of the urine donors and the timing of the urine sample collection have a bearing on the urinary miRNA and uEVs content, and
- investigate how the urinary miRNA profiles of patients with LUTD differ from controls.

To answer the first question, we recruited two groups of male volunteers (n = 6 per group) without LUT symptoms, “young” (mean age 32 ± 6.6 years old) and “old” (mean age 57 ± 6.1 years old). Approximately 100 ml of first-void and afternoon-void mid-stream urine samples were collected on the same day, and processed as described in “Methods”. The samples from each time point were divided into 2 × 37 ml fractions and used for (1) total cell-free urinary RNA isolation and (2) serial (ultra)centrifugation (UC), protease inhibition, DL-dithiothreitol (DTT) treatment, filtration and size-exclusion chromatography (SEC) to isolate uEVs for further analysis^27^.

To answer the second question, we isolated total RNA from urine samples of patients with LUTD (n = 38) and age-matched controls (n = 12) and investigated the expression profiles of 800 miRNAs using the NanoString nCounter Human miRNA Expression Assay kit followed by validation with RT-qPCR.

### Comparison of yields, size distribution and protein content of uEVs isolated from “young” and “old” subjects at different time points

**Figure 1:**
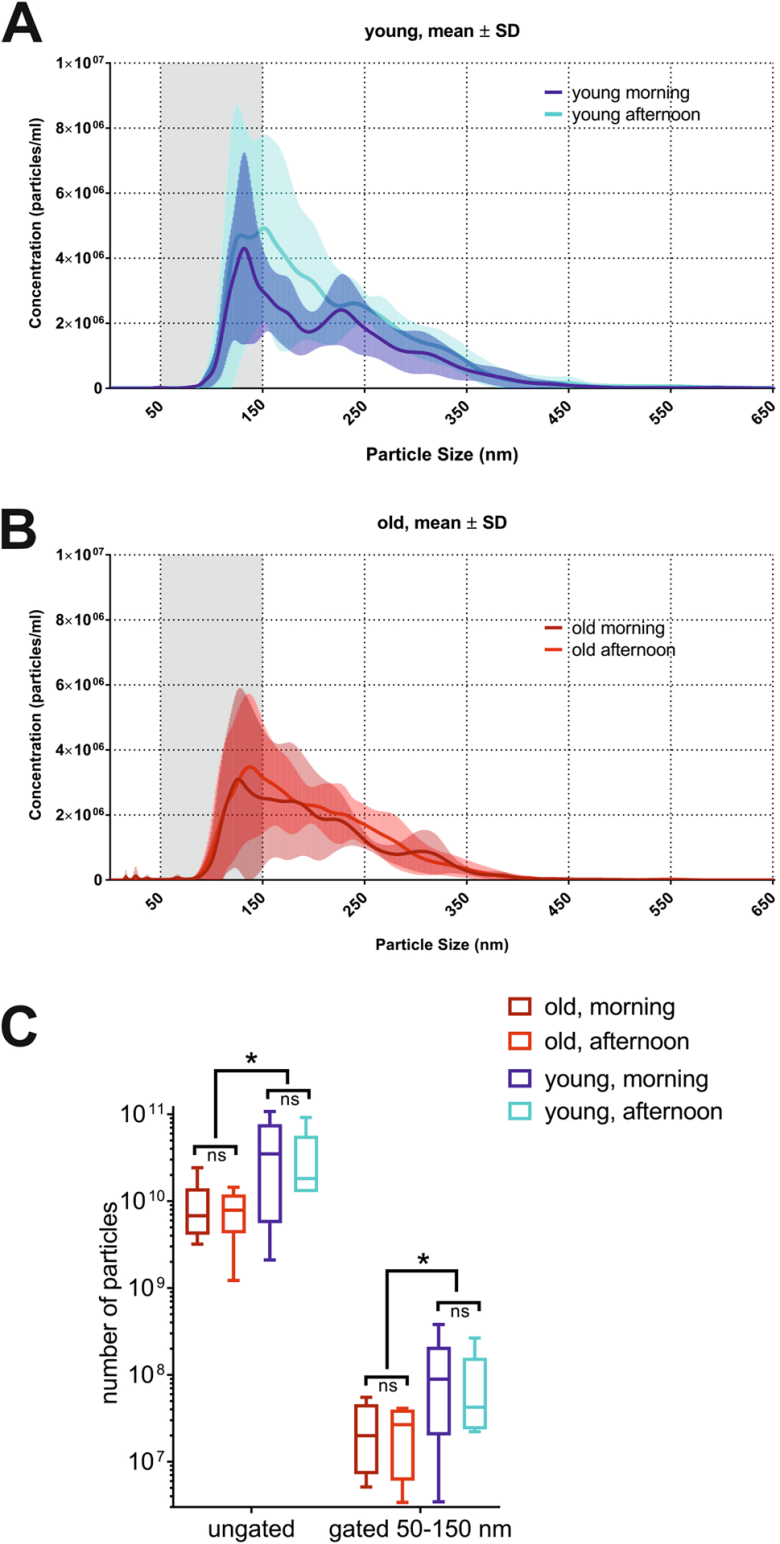
Particle yields of uEVs isolated from two age-matched groups of healthy volunteers at different time-points. uEVs were isolated by UC-SEC (ultracentrifugation followed by size exclusion chromatography) from 37 ml first-void urine (morning) and 37 ml void during the day (afternoon) of two groups of male healthy volunteers (n = 6 per group) without LUT symptoms: “young” (mean age 35 ± 6.6 years old) and “old” (mean age 57 ± 6.1 years old). Samples were eluted in the same volume to allow concentration comparison. **(A)** Number of uEVs isolated in “young” subjects at two time points of sample collection (morning and afternoon). NTA was performed on isolated uEVs to calculate total particle concentrations (Y-axis). Size distribution graphs show the concentration on Y-axis and size distribution in X-axis. Particles in range of 50–150 nm (shaded area) in the same sample are shown. The results are shown as mean ± SD of 6 samples per each urine collection time point. **(B)** Number of uEVs isolated in “old” subjects at two time points of sample collection (morning and afternoon). Size distribution graphs show the concentration on Y-axis and size distribution in X-axis. **(C)** Number of all particles and particles gated for size (between 50–150 nm) in each subjects peak content fraction was counted in “young” and “old” groups. No difference was observed in particle yields (gated and ungated) within an age group regarding collection time (paired, two-tailed t-test). Significant differences between the “young” and the “old” groups were observed in the total particle yields and in the yields of particles gated (50–150 nm) to exosome sizes (* p < 0.05, unpaired, two-tailed t-test).

Particles harvested from 37 ml of urine from the “young” and “old” subjects by UC-SEC were subjected to nanoparticle tracking analysis (NTA) using NanoSight NS300 as described in “Methods”. Total particle yield and the content of 50–150 nm uEVs, corresponding to the exosome population were estimated. The isolated amount of particles from the afternoon sample of one “young” subject was under the detection limit of NanoSight NS300, thus particles could not be counted in this sample. The size distribution of uEVs isolated from the morning and afternoon urine in the “young” group are comparable, ranging from 75 to 400 nm (Fig. 1A). The highest particle concentration was observed in the range between 50 and 150 nm, corresponding to the urinary exosomes (Fig. 1A). In the “old” subjects, the distribution of particle size in the morning and afternoon uEVs isolations was similar (Fig. 1B) and comparable to the “young” subjects. “Young” subjects yielded significantly more particles than the “old” in total (means 3.71E + 10 and 8.64E + 09 particles respectively, p-value = 0.0152 unpaired, two-tailed t-test) and gated to sizes 50–150 nm (means 1.04E + 08 and 2.46E + 07 particles, respectively, p-value = 0.0347 unpaired, two-tailed t-test) particle counts in uEVs preparations (Fig. 1C). Paired two-tailed t-test showed no significant difference in the number of uEVs between morning and afternoon urine within the same age group (Fig. 1C). After gating for the exosome size, the count of 50–150 nm uEVs in all samples was reduced (Fig. 1C), indicating that all preparations contained larger particle and/or protein aggregates in addition to exosomes. Overall the 50–150 nm exosome subpopulation in different UC-SEC preps was consistent between the subjects of each age group.

**Figure 2:**
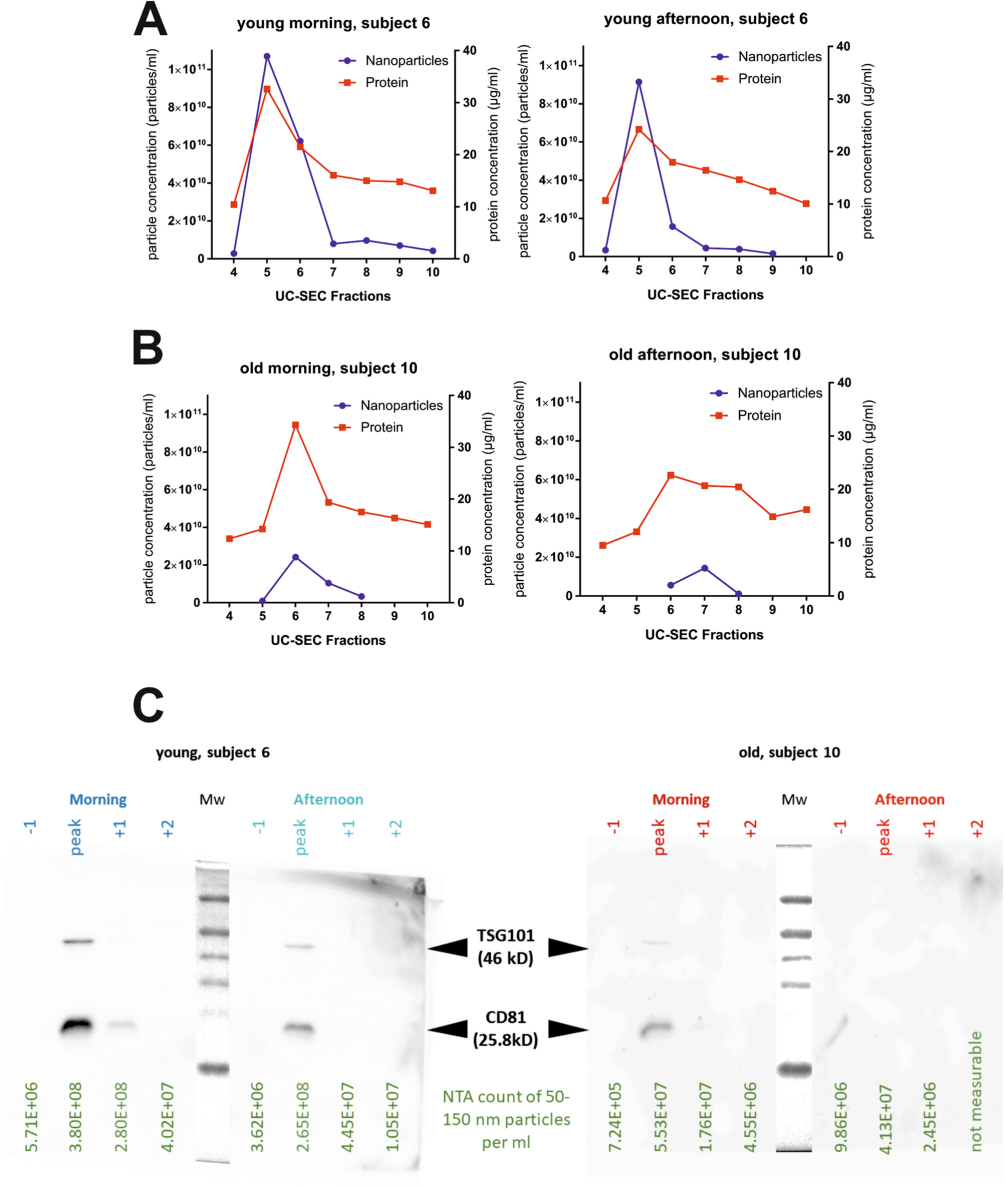
Exosomal markers and protein load in UC-SEC fractions from a young and an old representative sample. **(A)** Particle and protein concentrations in peak fractions of the UC-SEC of a young subject 6 (age 39 year old). The data for first-void urine uEVs isolation (left) and afternoon void urine insolation (right) are shown. **(B)** Particle and protein concentrations in peak fractions of the UC-SEC of an old subject 10 (age 59 year old). The data for first-void urine uEVs isolation (left) and afternoon void urine insolation (right) are shown. **(C)** Exosomal markers CD81 and TSG101 were tested by Western blotting in 27 µl of fractions including the peak of particle concentration determined by NTA from each isolation (subjects 6 and 10). NTA particle content for 50–150 nm exosomes of each lane indicated. Uncropped images of 2 blots (young subject 6 and old subject 10 with Mw markers) are presented.

Protein content was measured in the UC-SEC fractions, and, in contrast to the particle numbers, there was no difference between “old” and the “young” subjects at either time point (Supplementary Fig. S1). Taking into account a significant (p-value = 0.0347) difference in the exosome content between the age groups (Fig. 1C), we sought to investigate whether exosomal proteins were detected in fractions in accordance with the uEVs content. Representative results in two subjects are shown (Fig. 2). The peak protein concentration was measured in the same UC-SEC fraction in which the higher particle number was detected, in accordance with our earlier observations^27^ (Fig. 2A,B). In young subject 6 we recovered approximately 1.07e + 011 uEVs in the peak fraction of the morning and 9.14e + 010 uEVs of the afternoon prep (Fig. 2A), compared to 2.42e + 010 and 1.44e + 010 in older subject 10 (Fig. 2B). In contrast, the protein content of the UC-SEC fraction was comparable in both groups (morning peak of 32 and 34 µg/ml, afternoon peak 24 and 22 µg/ml in “young” and “old”, respectively).

To further characterize the uEVs, we subjected UC-SEC fractions to Western blot analysis with antibodies against exosomal marker proteins CD81 and TSG101 as described in “Methods”. In a representative experiment with the uEVs from one young and one old subject (Fig. 2C), TSG101 and CD81 were detected in the peak particle fraction of the morning and afternoon preps from subject 6 (“young”), but only in the peak particle fraction of the morning prep in subject 10 (“old”) in good agreement with NTA particle count (Fig. 2C, 50–150 nm gated particles loaded on each lane are shown below). When loading 27 µl samples per lane, approximately 5.5E + 06 particles per ml (size range 50–150 nm) were detectable with CD81 antibody. These findings indicate the contribution of the other, non-exosomal proteins, to the total protein content in UC-SEC fractions.

### Characterization of RNA load in total urine and uEVs isolated from the “young” and “old” subjects at different time points

**Figure 3:**
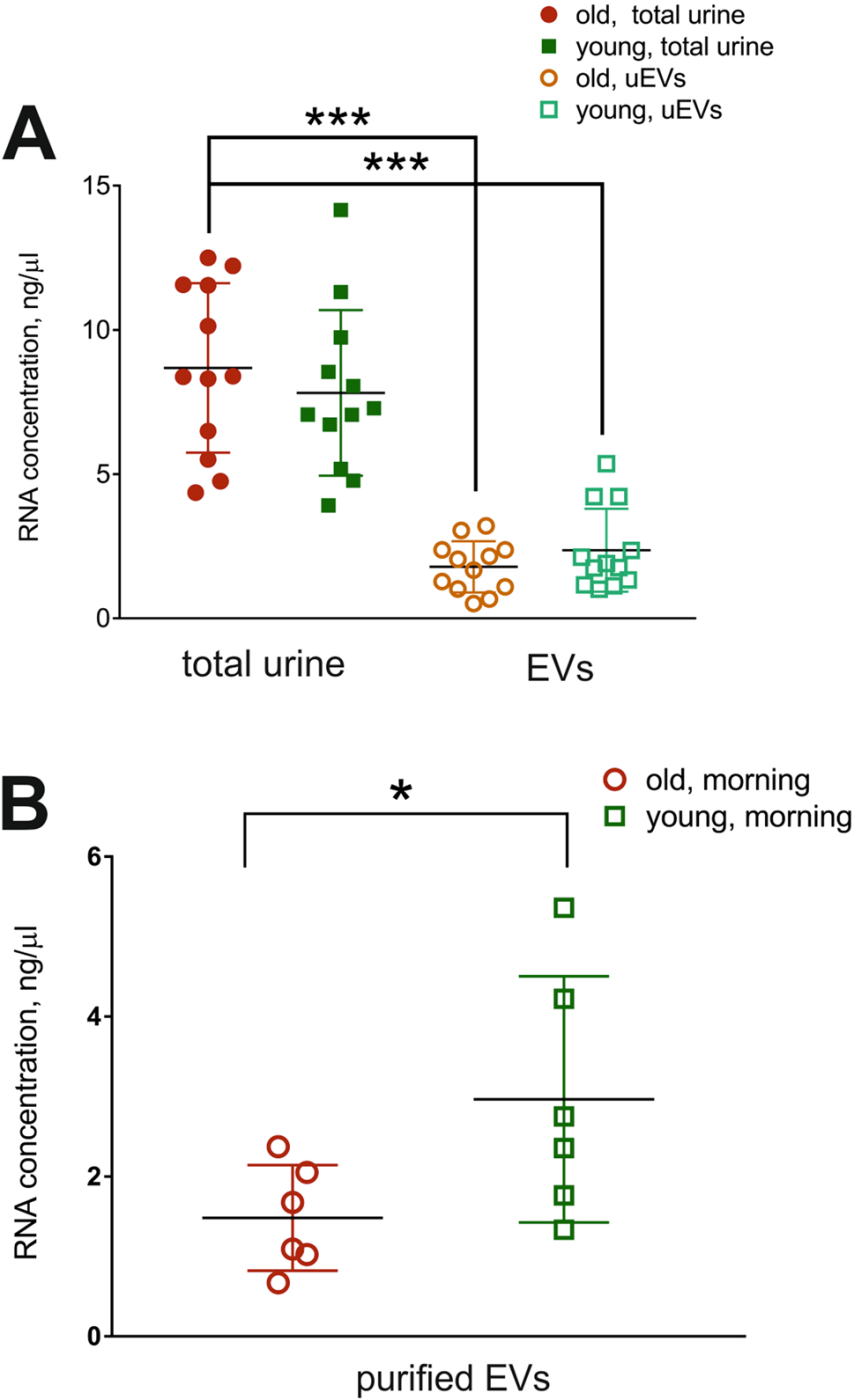
RNA yields from total urine and uEVs in “young” and “old” groups. **(A)** RNA from 37 ml of cell-free total urine and from uEVs isolated from the same urine volume were eluted in 50 µl of buffer and RNA concentration determined by PCR-based Low Abundance RNA Quantification (LA qPCR) Kit. Significantly lower RNA yields (*** p < 0.001, ANOVA) were observed when using purified uEVs compared to the total urinary RNA, containing both naked and packaged RNAs. **(B)** Yields of RNA isolated from uEVs present in the morning urine in the “young” group were higher, in agreement with the higher NTA particle counts (*p < 0.05, unpaired, two-tailed t-test).

We purified total cell-free RNA from urine and isolated RNA from uEVs isolated by UC-SEC using the same volume of urine collected from the same person at 2 time points (morning first void and afternoon void). UC-SEC fractions 5–10 showed the highest number of particles by NTA, thus they were pooled and RNA isolated. Overall the highest recovery of uEVs was achieved in the morning urine preps of the “young” subjects (means 1.04E + 08 and 2.46E + 07 particles, respectively, p-value = 0.0347, Fig. 1C). As previously described, total urine and urinary exosomal RNA contain mostly small RNAs^27,30^. The urinary exosome RNAs are predominantly 25 nt long and relatively enriched in miRNAs^30^, while total cell-free urinary RNA has a large proportion of YRNA and tRNA fragments, as well as other small RNA species^31^. RNA was isolated from the same starting amount of urine and eluted in the same volume, allowing the comparison of RNA concentrations between samples, proportional to the RNA yield. Because the recovered concentrations were at the limit of the spectrophotometer detection range, we used qPCR-based Low Abundance RNA Quantification Kit (Norgen). Overall, the total RNA yields did not vary between the morning and afternoon samples, and there was no difference between the “young” and “old” subjects (Fig. 3A). There was a significant difference (p = 0.0006, one-way ANOVA) between the amount of RNA recovered from the uEVs vs. total cell-free RNA from the same urine sample, indicating that not all RNAs are packaged in the vesicles (Fig. 3A). We compared the yields of RNA from uEVs, isolated from the “young” and “old” groups in the morning, when the number of particles was the greatest (Fig. 1C). There was a significant increase (p = 0.0452, unpaired, two-tailed t-test) in the RNA amounts, isolated from the young subjects, in agreement with the higher uEV input (Fig. 3B).

### miRNA profiling in total urine and urinary exosomes of age-matched healthy subjects

**Figure 4:**
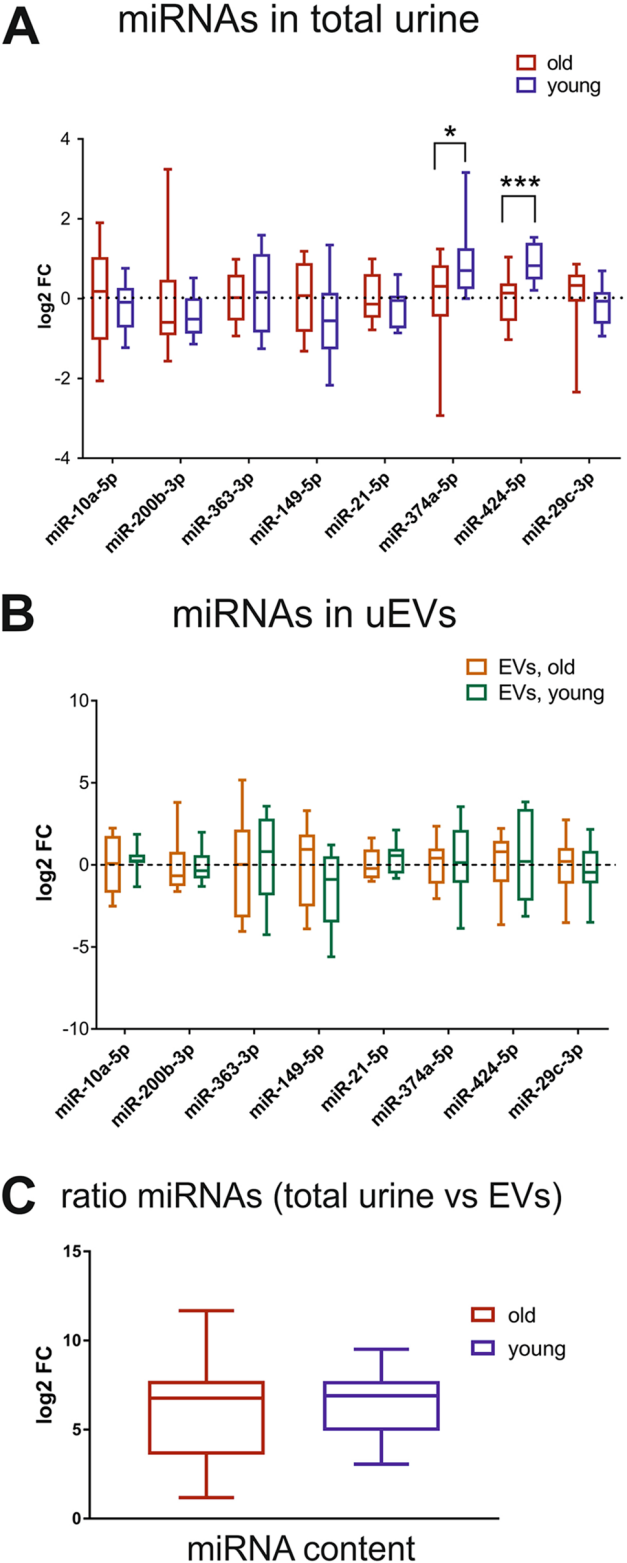
Profiling of abundant miRNA in the total urine and uEVs of healthy age-matched groups. RNA samples were derived directly from 37 ml of total cell-free urine or from uEVs, isolated by UC-SEC from 37 ml of the same urine sample, and miRNAs were profiled using RT-qPCR. **(A)** RT-qPCR results for 8 abundant miRNAs detected in the total urine of “young” and “old” healthy subjects in the morning and afternoon collection (n = 12 per group). Boxplot of log2 fold change relative to the average normalized Ct values in the “old” group. Statistically significant differences * p < 0.05, *** p < 0.001, multiple t-tests). **(B)** RT-qPCR results for 8 abundant miRNAs detected in the uEVs of “young” and “old” healthy subjects in the morning and afternoon urine (n = 12 per group). Boxplot of log2 fold change relative to “old” group. No significant differences were observed. **(C)** Log2 fold change between the total miRNA content, proportional to the average Ct values in all samples, and the exosomal miRNA content in the “old” and “young” subjects. No significant differences were observed.

We sought to investigate whether the age influences the composition of urinary miRNA in the cell-free urine or uEVs fraction in volunteers without LUTD. Urinary exosomes were purified from 37 ml urine by UC-SEC, and RNA isolated from the 5 UC-SEC fractions containing the most uEVs. In parallel, total RNA was isolated from 37 ml of the same urine sample. Based on our previous results^27^ and the recent NGS-based study of miRNA expression in the urine and other biofluids of young (18–25 year old) heathy volunteers^31^, we selected 8 abundant miRNAs and assayed their presence by RT-qPCR. All selected miRNAs were robustly expressed in the total urine samples from the “young” and “old” subjects of this study and showed no age-related differences, except miR-374a-5p and miR-424-5p which were significantly (p = 0.044 and 0.00049, respectively) higher in the younger subjects (Fig. 4A).

We compared the same miRNAs in the purified uEVs, and although there was some variation in the amounts detected in the “young” vs. “old” subjects, the differences were not significant (Fig. 4B). As expected, the exosomal contents of the selected miRNAs was lower than their amounts in the total urine, with the mean fold difference of 84 in “young” and 64 in “old” (Fig. 4C).

### Patient selection and grouping

Patients with LUTD were recruited in the study as described in “Methods”. Controls (n = 12) were mostly selected from patients with no obstruction, IPSS score < 8, a bell-shaped flow curve and no post-void residual urine. Average age of controls was 57 ± 6 years old. BLUTD group (n = 26) included patients with LUTD due to BOO characterized by increased detrusor pressure and reduced urine flow during pressure flow, defined as obstructed according to Abrams Griffiths nomogram^32^ with or without involuntary detrusor contractions during the filling phase (phasic and/or terminal detrusor overactivity, DO). BLUTD groups also contained patients with contractions of reduced strength and/or duration with a low flow rate, resulting in prolonged bladder emptying and / or failure to achieve complete bladder emptying within a normal time span or without detrusor contractions during the voiding phase (detrusor underactivity), and cystoscopy in line with obstruction. Average age of BLUTD patients was 74.2 ± 11 years old. NLUTD group (n = 12) were patients with LUTD caused by neurological diseases and spinal cord injury. Average age of NLUTD patients was 53.2 ± 17.1 years old. Prior to UDI an acute urinary tract infection was ruled out by dipstick testing.

### Patient urinary miRNA profiling and validation

**Figure 5:**
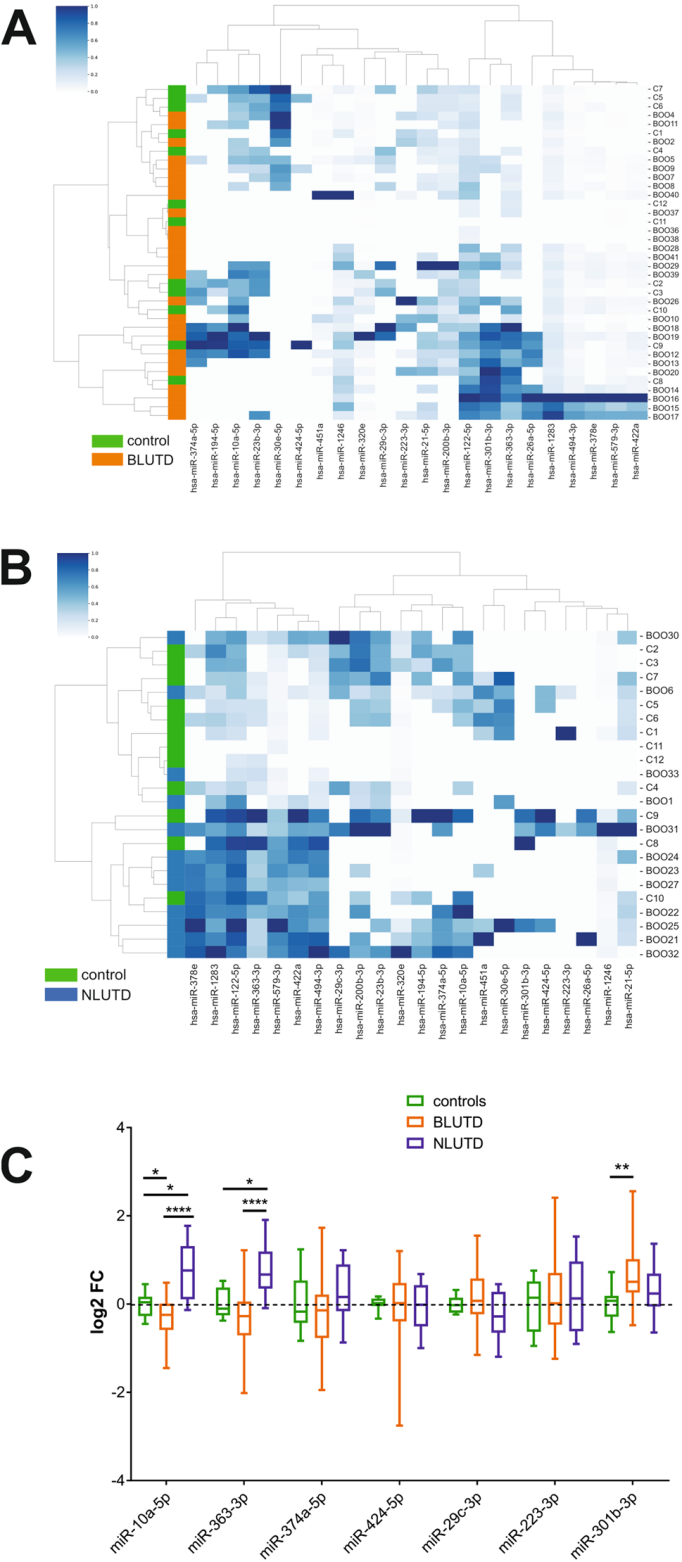
Urinary miRNAs miR-10a-5p, miR-363-3p and miR-301b-3p differentiate between controls and LUTD patients groups. RNA samples were derived directly from 50 ml of total cell-free urine, and miRNAs were profiled using NanoString and validated by RT-qPCR. **(A)** Hierarchical clustering and heatmap of 22 miRNAs expressed in the total urine of controls (“C”, n = 12) and patients with BLUTD (“BOO”, n = 26). Each column represents one miRNA and each row represents one sample. Expression levels are colour-coded (bar above, white for low and blue for high levels). Samples are colour-coded orange for BLUTD and green for control. **(B)** Hierarchical clustering and heatmap of 22 miRNAs expressed in the total urine of controls (“C”, n = 12) and patients with NLUTD (“BOO”, n = 12). Each column represents one miRNA and each row represents one sample. Expression levels are colour-coded (bar above, white for low and blue for high levels). Samples are colour-coded blue for NLUTD and green for control. **(C)** PCR validation for NanoString data for 7 regulated miRNAs, normalized to miR-320e. Boxplot of log2 fold change in patients with BLUTD (n = 30) and NLUTD (n = 11) relative to controls (n = 12) Ordinary 2 way ANOVA was used for multiple comparisons (*p ≤ 0.05, ** p = 0.0047, **** p < 0.0001).

Following total RNA isolation from the urine of patients with LUTD and controls, we performed profiling of a panel of 800 human miRNAs using the NanoString nCounter Assay kit as described in “Methods”. We could detect 40 miRNAs in at least 25 samples, and 321 miRNAs in at least 2 out of 50 total samples after normalization (Supplementary file “Patients_Nanostring_data_norm.xlsx"). Using expression profiles of 22 abundant urinary miRNAs we performed hierarchical clustering analysis of the BLUTD group and controls. Heatmap shows that BLUTD samples clustered in 2 main groups outside controls (Fig. 5A). We performed a similar analysis using urinary RNA from NLUTD samples. Here as well the patients’ samples mostly clustered apart from controls (Fig. 5B). NanoString results showed regulation of miRNAs miR-301b-3p, miR-363-3p and several other miRNAs relevant in BLUTD^26^, prompting us to validate these by RT-qPCR in a larger patients’ cohort.

MiR-320e was robustly detected in all samples, and previously identified as a prevalent urinary miRNA, not packaged into uEVs^27^. We tested its expression in the panel of “young” and “old” control subjects, and observed no age-induced variation of its urinary levels (Supplementary Fig. S2A). We also assessed the correlation between Ct values for miR-320e, the sample average Ct values, which are often used to normalize RT-qPCR results, and the RNA concentration in the same sample. There was a good positive correlation with the average Ct values, and expected negative correlation between Ct values of miR-320e and RNA concentrations (Fig. S2B), indicating that miR-320e can be used as a normalizer of RT-qPCR results in total urine samples. The Ct values for miR-320e were similar in all sample groups (Fig. S2C), and the expression levels of miR-320e in the BLUTD and NLUTD groups were not different from the controls (Fig. S2D), confirming that normalizing the results to miR-320e would not distort the expression values of the other miRNAs. Previously, miR-301b-3p was not reported in the young subjects’ urine neither in the urinary NGS profiles^31^, nor in our earlier NanoString study^27^. Therefore, in order to verify the NanoString results we first tested miR-301b-3p expression in the control samples by RT-qPCR (supplementary Fig. S3). Mir-301b-3p was detected by PCR in all controls, and its levels were slightly elevated in the “young” group, though this difference was not significant (Fig. S3). RT-qPCR validation of seven selected miRNAs showed a significant (p = 0.0046) up-regulation of miR-301b-3p in BLUTD compared to controls, and a down-regulation (p = 0.039) of miR-10a-5p (Fig. 5C). In contrast, both miR-10a-5p and miR-363-3p were significantly (p = 0.0223 and p = 0.0127, respectively) up-regulated in the urine of patients with NLUTD (Fig. 5C) compared to controls. In NLUTD miR-301b-3p showed a tendency for up-regulation, but the result was not significant, possibly due to the smaller samples size (12 NLUTD vs. 30 BLUTD). The opposite regulation of miR-363-3p and miR-10a-5p in the BLUTD and NLUTD groups was confirmed by two-way ANOVA (p < 0.0001) (Fig. 5C).

### A combination three urinary miRNA signature (miR-10a-5p, miR-301b-3p and miR-363-3p) discriminates between BLUTD, NLUTD and control

**Figure 6:**
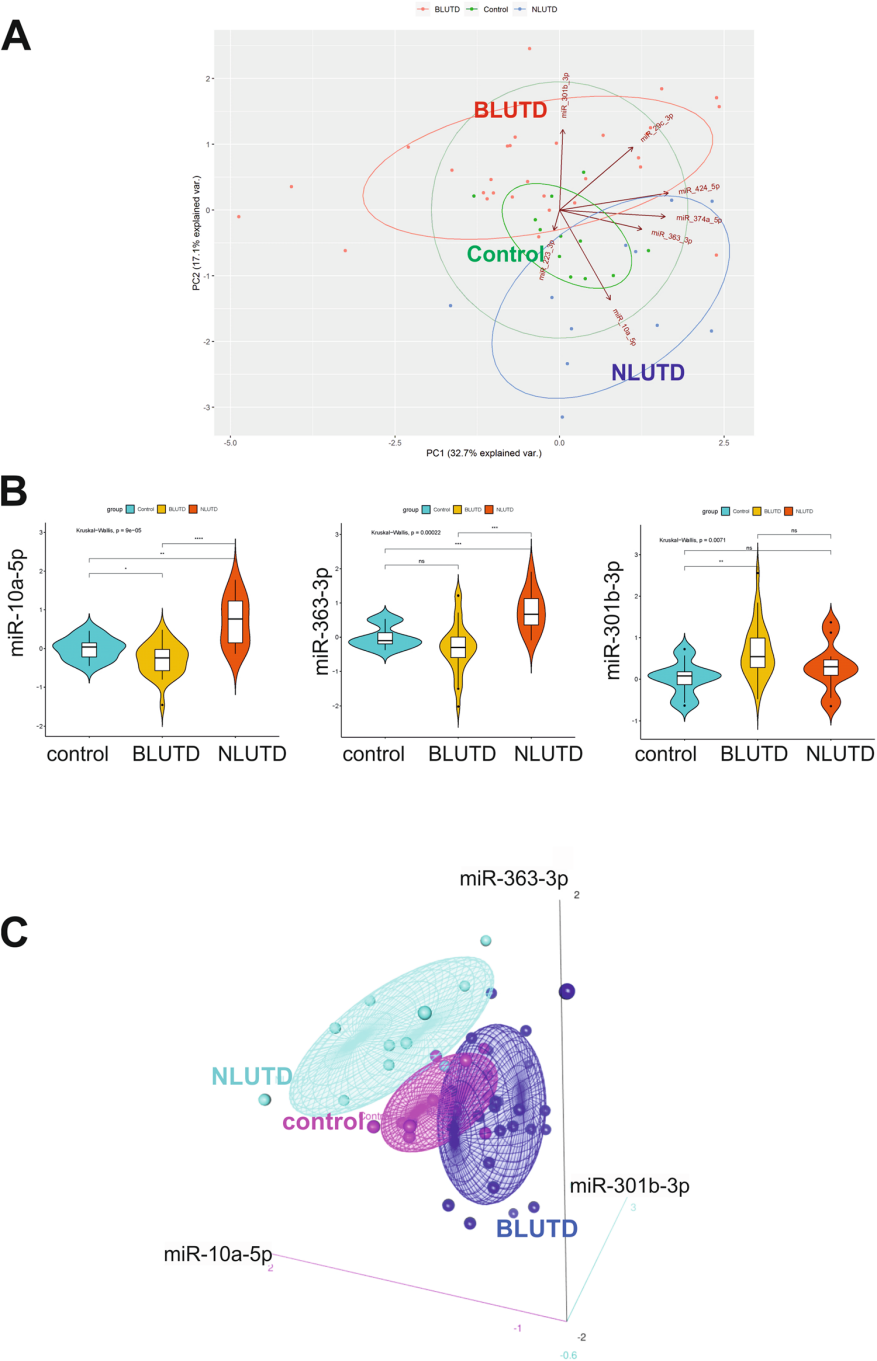
Identification of a three miRNA urinary biomarker signature miR-10a-5p, miR-363-3p and miR-301b-3p for obstruction-induced LUTD. **(A)** Principal component analysis of miRNA expression was done based on profiles of 7 miRNAs determined by RT-qPCR for each group (n = 12 controls, n = 30 BLUTD and n = 11 NLUTD). The position of patients from each group on the plot indicates the similarity of miRNA expression pattern. Controls are shown in green, NLUTD in blue, BLUTD in red. PCA confirms that NLUTD samples are well separated from BLUTD based on their miRNA profiles. In this figure, the loadings are represented by arrows. Axis PC2 has a strong negative loading for mir-10a-5p, and strong positive loadings for mir-301b-3b. Axis PC1 shows a strong positive loading for mir-363-3p. **(B)** Regulation of the 3 significantly changed miRNAs in urinary samples of controls and patients with BLUTD and NLUTD. RT-qPCR results in patients’ groups are shown in a violin plot as log2 fold changes compared to controls. The graph is representing minimum, first quartile, median, third quartile, and maximum. *p < 0.05 **p < 0.001 and ***p < 0.0001 (Kruskal–Wallis test). **(C)** Three-dimensional scatterplots and point identification for 3 miRNA urinary biomarkers for bladder outlet obstruction. Log2 fold change values of miRNAs, miR-10a-5p, miR-301b-3p and miR-363-3p suggested by RT-qPCR validation of NanoString miRNA profiling of urinary miRNAs of patients with obstructive or neurogenic LUTD and controls are plotted. BLUTD values are shown in dark blue, NLUTD values in light blue and control values in pink. The three-miRNA signatures are sufficient to discriminate BLUTD, NLUTD and control groups from each other.

The principal component analysis (PCA) was performed using log2 fold changes of the seven miRNAs validated by RT-qPCR (Fig. 6A). The fold change was calculated as the ratio of the normalized Ct values of BLUTD and NLUTD related to the normalized Ct values in controls. These values then were log2 transformed. The opposite regulation of miR-301b-3p and miR-10a-5p–miR-363-3p pair allowed to separate the NLUTD from BLUTD groups, with control values falling in between. The arrows in Fig. 6A represent the loadings, analogous by magnitude with the covariances/correlations observed between the variables in the covariance matrix. The lengths and directions of loadings for miR-301b-3p, miR-10a-5p and miR-363-3p indicate their strong contribution to the principal components. MicroRNA miR-10a-5p was significantly down-regulated in BLUTD, but up-regulated in NLUTD group, compared to the controls, with significant difference between the two patients’ groups (Kruskal–Wallis test, p = 9E−05) (Fig. 6B). Similarly, miR-363-3p was up-regulated in NLUTD group, compared to the BLUTD and controls (p = 0.00022), whereas miR-301b-3p was higher in BLUTD (p = 0.0071). These data indicate that the urinary miRNA profiles could discriminate between controls and LUTD, and obstructed and neurogenic LUTD groups from each other. Log2 fold change values for miRNAs miR-301b-3p, miR10a-5p and miR-363-3p in the urine of 12 controls, 30 BLUTD and 11 NLUTD are illustrated in a 3D scatter plots (Fig. 6C). The coordinates of each sample correspond to the log2 fold changes of the 3 miRNA markers, and the confidence ellipse represents an iso-contour of the Gaussian distribution, and allows visualization of a 3D confidence interval (75%).

## Discussion

Lower urinary tract dysfunction (LUTD) results from various diseases including bladder outlet obstruction (BOO) and neurologic disorders^33^. LUTD results in structural and functional alterations in the bladder wall, reflected in the changes of mRNAs and miRNAs gene expression profiles^26^. These changes, initially observed by analysing detrusor biopsy material, could be detectable in patients’ urine, because the circulating miRNAs are abundant in biofluids, where RNA binding proteins or packaging in microvesicles protects them from degradation. Indeed, it has been shown that miRNAs are relatively stable in urine under a variety of storage conditions, which supports their utility as urinary biomarkers^34^. In this study, we sought to identify a panel of urinary miRNAs, specific for LUTD.

Age-matching of controls is very important while investigating urinary biomarkers because urine metabolome changes with age^35^. Less is known about the effect of ageing on biogenesis and release of extracellular vesicles (EVs), though such differences have been observed in many diseases^36^. Importantly, differences in microvesicle amounts and their miRNA cargo have been reported during ageing and senescence in cultured cells^37^, animal models^38^ and human diseases such as diabetes^39^ and neurodegenerative diseases^40^.

Taking into account the advanced age of patients with LUTD, particularly men with benign prostatic obstruction (BPO), we aimed to assess the influence of age and timing of urine collection on urinary EVs (uEVs) release and resulting miRNA content, in order to provide appropriate controls for biomarker studies. We wanted to take into consideration both miRNAs from the total urine, and those packaged in uEVs. Urine was collected at different time points from volunteers with a significant age difference (mean age difference 22 years) in order to study the number of uEVs and miRNA profiles in total RNA and RNA packaged in uEVs. We demonstrated that urine samples from the “young” contained significantly more uEVs than urine from the “old”. Similarly, more exosomes could be isolated in the “young”, judged by size distribution and the presence of exosomal markers. Timing of urine collection (first-void in the morning, or an afternoon void) did not have a significant impact on the amount of uEVs or the RNA content of urine, including miRNAs. These findings are consistent with previous observations showing that despite significant sex-related differences in urinary miRNAs, the exact timing of urine collection has minor consequences for miRNA composition of samples^30^. In the present study, there were no significant age-related differences in the total amount of proteins and RNA, but we observed a slight increase in RNA packaged in uEVs in the young.

We profiled the expression of 8 miRNAs, abundant and robustly detected in the urine and uEVs of subjects without LUTD in earlier studies^27,31^. There was no significant difference in exosomal miRNAs between the two age groups, however, we observed a significant increase in miR-374a-5p and miR-424-5p in the total RNA of the young. MiR-374a-5p plays a regulatory role in cell growth and differentiation, and calcium handling. It is dysregulated in many diseases, including a marked decrease in myocardial ischemia‐reperfusion (reviewed in^41^). Likewise, anti-inflammatory miR-424-5p promotes cell differentiation^42^ and is down-regulated in many cancers^43^. As expected, considerably fewer miRNAs could be detected in uEVs vs. total urine. Therefore, taking into consideration that a relatively small volume of urine was collected from the patients with LUTD before UDI (up to 50 ml), we decided to investigate the miRNA profiles in the total cell-free urine rather than in the exosomal fraction.

Only male controls were selected and age matching was done to the best of our ability. While the mean age of controls (57 ± 6 year old) was close to the NLUTD group (53.2 ± 17.1 year old), it was still lower than in the BLUTD group (74.2 ± 11 year old). Because the incidence of BPO driven by benign prostatic enlargement increases with age, it is virtually impossible to find perfectly age matched controls without LUTS for this group. Initial miRNA screening using the Nanostring nCounter platform showed that the patient groups could be separated by hierarchical clustering according to 22 robustly detected differentially expressed miRNAs. Urinary miR-320e, which is not packaged in uEVs^27^, was most abundantly detected in all samples. We evaluated its correlation with the input RNA amounts, and confirmed that it can be used as a reliable normalizer in RT-qPCR and its amounts are stable in different age groups.

Validation of the NanoString data by RT-qPCR demonstrated that miRNA miR-10a-5p was significantly lower in BPO-induced LUTD than in controls, while miR-301b-3p was significantly up-regulated. MiR-363-3p was reduced in BLUTD group, though not significantly. Interestingly, in NLUTD samples, miR-10a-5p and miR-363-3p showed the opposite regulation compared to the BLUTD group: both miR-10a-5p and miR-363-3p were highly increased. Up-regulation of miR-301b-3p was not significant in NLUTD. Discriminative behavior of miR-10a-5p in NLUTD vs. BLUTD was interesting in view of our previous study in biopsies from patients with BLUTD^26^, where this miRNA was a part of a three miRNA signature (hsa-miR-103a-3p, hsa-miR10a-5p and hsa-miR-199a-3p) of the bladder functional phenotypes. MiR-301b-3p was increased in all patients with LUTD. This miRNA is abundant in the plasma, but not in the urine of the young controls^27,31^, though one study detected it in the urine from patients with bladder cancer^44^. Here we show that miR-301b-3p can be reliably amplified from the urine of both young and older controls. MiR-301b-3p is induced by hypoxia^45^, which is common in BOO^46^. Its up-regulation in both groups with LUTD (highly significant in BLUTD, an elevating trend in NLUTD group) might be indicative of BOO. We propose a urinary biomarker signature for the obstruction-induced LUTD (miR-10a-5p, miR-301b-3p and miR-363-3p) and are currently testing it in a larger sample cohort.

In summary, our study provides the first evidence of the age-related differences in the number of secreted uEVs and in miRNA composition between the total urine samples from young and older controls. This further strengthens the necessity of matching controls to the test subjects by age, whereas the timing of urine collection is less important for uEVs and miRNA analysis. RT-qPCR is a method of choice for the patients’ screening, and here we propose using an abundant urinary miR-320e as a normalizer because its Ct values show a strong inverse correlation with the RNA input. MiRNAs miR-10a-5p, miR-301b-3p and miR-363-3p could be further investigated as urinary LUTD biomarkers, offering a non-invasive tool for the detection of BOO.

## Methods

### Study approval

Permission to conduct this study was obtained from the Ethics Committee of Canton Bern, Switzerland (KEK 2017–01609), and all subjects gave written informed consent. The methods in this study were carried out in accordance with the approved guidelines by University of Bern and University Hospital of Bern and all experimental protocols were approved by the ethics committee.

### Sample collection

#### Healthy volunteers

Twelve male subjects without LUTS were recruited in the first part of this study, and separated into two groups (n = 6 persons each)—“young” (mean age 32 ± 6.6 years old) and “old” (mean age 57 ± 6.1 years old). Approximately 100 ml of both first-void and afternoon-void mid-stream urine were collected on the same day directly into sterile 50 ml plastic centrifuge tubes (Starstedt, Switzerland) and stored at 4 °C no longer than 4 h before being further processed.

#### LUTD patients

Patients with LUTD (n = 38), undergoing urodynamic investigation (UDI) at the Department of Urology, University Hospital Bern, submitted a urine sample (20–100 ml) immediately before the UDI. Samples were collected, processed by urine test and centrifugation as described below, and stored frozen at − 80 °C until RNA isolation.

#### Controls

(n = 12) urine samples were recruited from male patients who had IPSS score < 8, a bell-shaped flow curve and no post-void residual urine.

### Urine test

Urine was tested by dipstick to exclude infection (Combur 5 Test HC (Roche Diagnostics, Switzerland)). Only samples without signs of infection were included.

### Sample processing

In controls (“young” and “old” groups) 37 ml of morning and afternoon urine, respectively, were processed for total urine RNA isolation, and from another 37 ml of sample the uEVs were isolated, counted and used for Western blot analysis and RNA isolation. Freshly collected samples were centrifuged at 200 *g* at 4 °C for 10 min, then the cell-free supernatant was centrifuged at 2000 *g* at 4 °C for 10 min to discard the pellet of cell debris, bacteria and protein aggregates. Supernatant was then frozen and stored at − 80 °C until RNA isolation. Urine samples for exosome isolation were centrifuged at 200 *g* followed by 2000 *g*, both at for 4 °C for 20 min before being frozen and stored at − 80 °C. In LUTD patients, the urine samples (30–50 ml) were cleared by centrifugation as above and stored frozen at − 80 °C for total RNA isolation (below).

### uEVs isolation from morning and afternoon urine samples of age-matched controls

We used our established protocol of ultracentrifugation combined with size-exclusion chromatography (UC-SEC)^27^ to isolate uEVs from the first-void (morning) and afternoon urine samples (37 ml each) from the “young” and “old” controls. Briefly, frozen urine samples for exosome isolation were thawed at 4 °C overnight. To each 50 ml urine we added 4.2 ml protease inhibitor cocktail containing 6.875 mg 4-(2-Aminoethyl) benzenesulfonyl fluoride hydrochloride (AEBSF) (Sigma-Aldrich, St. Louis, MO, USA), and 50 µl Leupeptine (1 mg/ml in H_2_O) (Sigma-Aldrich, St. Louis, MO, USA). 36.5 ml of samples were further processed, starting with a 16,000 *g* centrifugation at 4 °C for 20 min. The supernatant was transferred in a fresh tube and stored at 4 °C while the pellet was treated with DL-dithiothreitol (DTT) (1 ml of 200 mg/ml, Sigma-Aldrich, St. Louis, MO) for 10 min at 37 °C to depolymerize Tamm-Horsfall Protein for higher uEVs yield. Then DL-DTT treated pellet and the stored supernatant was mixed together. Following centrifugation (16,000 *g*, 4 °C, 20 min) the pellet was discarded and the supernatant filtered with a 0.22 µm filter (Merck & Millipore). The filtrate was ultracentrifuged at 220,000 *g* in a fixed angle rotor (Beckman Coulter, Rotor Type 45 Ti, Optima L-90 K Ultracentrifuge) for 70 min at 4 °C to pellet the extracellular vesicles. The supernatant was discarded and the pellet containing uEVs was resuspended in 200 µl of particle-free PBS (Gibco) (UC sample).

Size exclusion chromatography (SEC) was performed using 2% cross-linked agarose gel filtration media (Sepharose CL-2B, GE Healthcare, 17014001, sepharose column height = 13 cm). A porous frit was positioned at the top of the Sepharose to avoid its disturbance during sample loading and elution. UC pellet sample was loaded onto the column and 25 fractions of 0.5 ml each were collected.

### Estimation of protein concentration

Protein concentration of samples was assessed after UC-SEC by Micro BCA Protein Assay Kit (Thermo Scientific) following manufacturer’s instructions except that the samples and BSA standards were measured in triplicates in volumes of 15 µl, 10 µl and 5 µl which were added to 150 µl of the kit’s working reagent.

### SDS-PAGE and western blot analysis

UC-SEC samples were solubilized in 4 × sample buffer and subjected to SDS-PAGE and Western blotting. Equal volume of sample (27 µl/lane) was loaded to compare the yields between preps. The primary antibody anti-CD81 (Santa Cruz, mouse monoclonal, Sc-166028) was applied with the dilution 1:500, anti-TSG101 (Abcam, mouse monoclonal, Ab83) with 1:1000 and the secondary anti-mouse HRP conjugated ECL antibody (GE Healthcare Life Sciences, NA031) with the dilution 1:1000.

### Nanoparticle tracking analysis (NTA)

For each UC and UC-SEC sample, nanoparticle size distribution, refractive index, the relative nanoparticle concentration of a particular size and the cumulative percentage of nanoparticles were investigated using NanoSight NS300 Instrument (Malvern Instruments Inc.) as described previously^27^. A suitable sample dilution in particle-free PBS (pH 7.2—7.4) was assessed by serial dilution and measurement to reach the reproducibility requirements (particles per frame and centers per frame between 10 and 100 with a deviation smaller than 2, and a concentration of particles below 1.0E + 010 to ensure measurements within detection range). Each experiment was performed in quintuplicate with static captures of 60 s duration. The capture and data analysis script (SETTEMP 25; CAMERAON; CAMERAGAIN 13; CAMERALEVEL 12; REPEATSTART; SYRINGELOAD 100; DELAY 10; SYRINGESTOP; DELAY 15; CAPTURE 60; DELAY 1; REPEAT 4; SETTEMP OFF; PROCESSSINGELSETTING; EXPORTRESULTS) was run using NTA 2.3 software.

### RNA isolation from total urine

The Norgen Biotek’s Urine Cell-Free Circulating RNA Purification Maxi Kit (Cat. 56600) was used to isolate miRNAs from up to 50 ml urine samples according to manufacturer’s instructions.

### miRNA isolation from exosomes

UC-SEC fractions 5–10 from each sample, which contained the peak concentration of nanoparticles, were pooled and RNA was isolated. First, exosomes were lysed with 0.5 volumes of 2× Denaturing Solution (Life Technologies, mirVana Paris Kit). Thereafter, RNA was isolated using mirVana miRNA Isolation Kit (Life Technologies) following the manufacturers instruction from chapter “E. Organic Extraction” starting with homogenizing the lysate with 1/10 volumes of miRNA Homogenate Additive.

### Assessing RNA quantity and quality

RNA concentration and quality were assessed using Nanodrop 1000 spectrophotometer (Thermo Scientific), and Low Abundance RNA Quantification (LA qPCR) kit (Norgen). After cDNA synthesis with LA qPCR kit, real-time PCR (RT-qPCR) was run using QuantStudio 3 Real-Time PCR System (ThermoFisher Scientific, Applied Biosystems). For the samples used in NanoString assays, Qubit fluorometer (Life Technologies), Qubit DNA BR Assay kit and Qubit Protein Assay kits were employed.

### cDNA synthesis and RT-qPCR

TaqMan Advanced miRNA cDNA Synthesis Kit (Applied Biosystems, A28007) was used according to manufacturer’s instructions. MicroRNA Advanced TaqMan assays were used for RT-qPCR and results analysed with web browser-based Applied Biosystems Real-Time PCR Analysis Modules. The Advanced miRNA TaqMan Assays do not include controls suitable for input cDNA normalization, therefore the individual miRNAs were normalized by subtracting from their Ct values the average Ct value of all miRNAs tested in the same sample. Alternatively, after the validation testing described in the Results (Supplementary Fig. S2), the Ct values of miR-320e were used as normalizer. For comparison between groups, the results were calculated as fold change ratios of the normalized Ct values for each miRNAs vs. the average normalized value of this miRNA in the control group. The fold change values were then log2 transformed.

### NanoString nCounter analysis

miRNA presence and content were analysed with the nCounter Human miRNA Expression Assay kit (NanoString, Seattle, WA) according to manufacturer's instructions with some modifications, as described in our earlier publication^27^. Briefly, 3 µl of each total RNA sample was used as input into the nCounter Human miRNA sample preparation. Hybridization was conducted for 12 h at 65 °C. Subsequently, the strip tubes were placed into the nCounter Prep Station for automated sample purification and subsequent reporter capture. Each sample was scanned for 600 FOV on the nCounter Digital Analyzer. The R Packages were used for NanoString Data Analysis: “NanoStringNorm” and “NanoStringDiff" (Available in CRAN). Background correction was performed based on the detected values of negative control probes, a within-sample normalization was calculated based on the observed values of positive control probes and normalization across samples using reference (housekeeping) genes. Additionally, we used a new algorithm called Removing Unwanted Variation-III (RUV-III)^47^ which employs technical replicates and suitable control genes to normalise the data. “EdgeR" was used for expression profiling. We also used inbuilt R commands for heatmap generation.

### Statistical analysis

Hierarchical clustering and the associated heatmaps for miRNA profiling data was generated with the *clustermap* function of python package *seaborn*. To find out the homogenous samples called clusters, based on their gene expression profiles, a hierarchically clustered heatmap has been plotted using Euclidean distance between samples. To choose reference (endogenous control) miRNA, we examined the correlation (Spearman) between the average Ct values across samples, the Ct value of the selected miRNA normalizer and the input RNA concentration. Data matrix was expressed as a correlation plot with values above ± 0.5 considered high.

To test the normality of the miRNA expression data we performed a q–q and Shapiro–Wilk’s normality tests to evaluate whether data meet the assumption of homogeneity of variances. For gene expression analysis the Student’s t-test or the analysis of variance (ANOVA) (one or two-way) was used to determine statistically significant differences between the means of two or more independent groups. Tukey's multiple comparisons and Sidak's multiple comparisons tests were used to correct P values. When the data did not present equal variance on the scores across the groups and could not meet the assumptions of ANOVA test, we used Kruskal–Wallis test. The P value < 0.05 was considered as statistically significant (GraphPad Prism (version 7.0a)).

## Supplementary Information

Supplementary Information.

Supplementary Figures.
