# Supplementary material for: Urinary miRNA profiles discriminate between obstruction-induced bladder dysfunction and healthy controls
Source: Sci Rep. 2021 May 13;11:10204. doi: 10.1038/s41598-021-89535-3 (PMC8119692; doi:10.1038/s41598-021-89535-3)
Supplement: Supplementary file 2 — Supplementary Figures. [file 41598_2021_89535_MOESM2_ESM.pdf]

# Supplementary Material

**To**

# "Urinary miRNA profiles discriminate between obstruction-induced bladder dysfunction and healthy controls"

**by**

Michelle von Siebenthal [1], Mustafa Besic[1], Ali Hashemi Gheinani [2,3], Akshay Akshay [1], Salomé Lizun-Platoni [3], Nadine Kunz[4], Fiona C. Burkhard [1, 4] and Katia Monastyrskaya [1, 4, *]

[1] Urology Research Laboratory, Department for BioMedical Research, University of Bern, Switzerland

[2] Urological Diseases Research Center, Boston Children's Hospital, Harvard Medical School, Boston, USA

[3] Broad Institute of MIT and Harvard, Cambridge, MA, USA

[4] Department of Urology, University Hospital, 3010 Bern, Switzerland

* Corresponding author:

K. Monastyrskaya - Urology Research Laboratory, Department for BioMedical Research, University of Bern, Switzerland Tel. +41 31 632 87 76; Fax: +41 31 632 05 51

E-mail address: katia.monastyrskaia@dbmr.unibe.ch

**Supplementary Figure S1. Protein content of UC-SEC fractions of "young" and "old" samples**

Protein content was measured in the SEC fractions of uEVs preparations in the "young" and "old" sample groups using the urine collected at different time points. (A) Protein content in the first-void urine isolations. (B) Protein content in the afternoon urine. No difference was observed between the preparations form the "old" and the "young" subjects at either time point.

**Supplementary Figure S2. Abundant urinary miRNA miR-320e can serve as a normalizer in RT-qPCR studies of urinary miRNAs**

(A) RNA was isolated from the total morning urine of healthy subjects and levels of miR-320e compared. Graph is a box whisker plot of log2 fold changes in "young" compared to the "old" group (n = 6 each).

(B) Spearman correlation plot of the miR-320e Ct, average sample Ct and known sample RNA concentrations (healthy volunteers' total urinary RNA, morning isolation, n = 12). There is a strong positive correlation between the Ct values or miR320e and average samlpe Ct, often used as normalizer, and a strong negative correlation between miR-320e Ct and sample RNA concentration. Correlation coefficient of -0.87 shows that miR-320e values determined by RT-qPCR are in good agreement with the total RNA input.

(C) Comparison of the average Ct values of all tested miRNAs (n = 8) in each sample and the Ct values for miR-320e. The groups are controls (n = 12), patients with BLUTD (n = 30) and patients with NLUTD (n = 11).

(D) miR-320e is not regulated in the LUTD patients. miR-320e was detected along with the other 7 miRNAs by RT-qPCR in the total urine of controls (n = 12), patients with BLUTD (n = 30) and patients with NLUTD (n = 11). After miR-320e Ct values were normalized by subtracting the sample average Ct values, the log2 fold differences to the control were calculated. No significant differences were detected.

**Supplementary Figure S3. miR-301b-3p is detected in the total urine of control subjects**

Cell-free RNA was isolated, and RT-qPCR was performed to detect miR-301b-3p in the total urine of healthy subjects. Log2 fold difference of the values in "young" group compared to the "old" group are shown in a box whisker plot. There was an elevation of miR-301b-3p levels in young subjects, but differences were not significant.

**Supplementary Figure S4. Gel images used in Fig.2**

Originals of the blots used in Fig.2, the same images taken at different exposures are inclused for comparison. The molecular weight ladder in the lower images is placed at 2 locations to show the integrity of the blot.

A

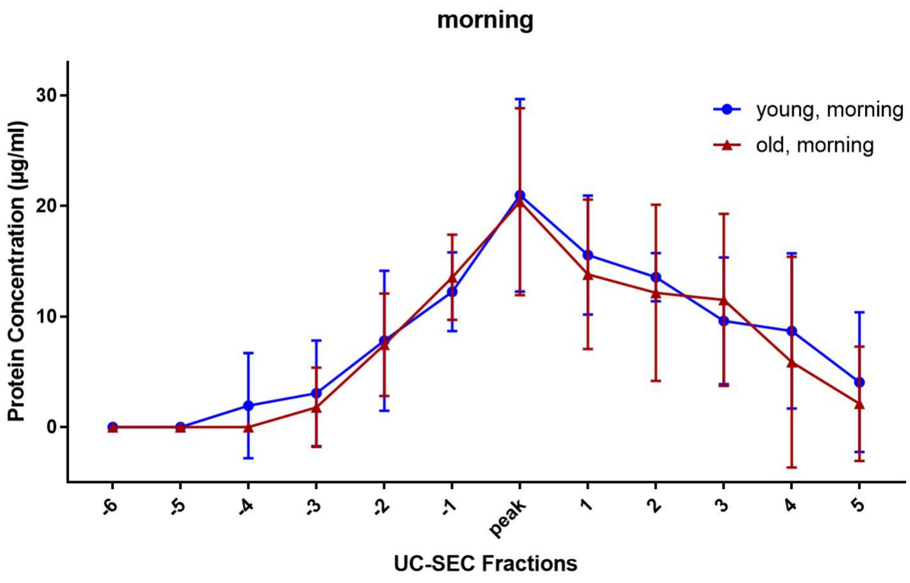
morning
Protein Concentration (µg/ml)
30
20
10
0
young, morning
old, morning
-6
-5
-4
-3
-2
-1
peak
1
2
3
4
5
UC-SEC Fractions

B

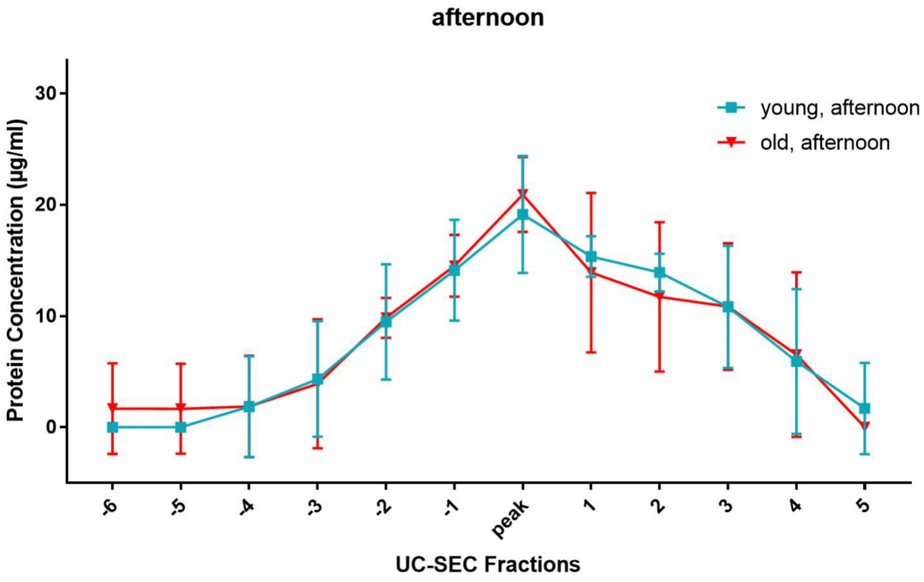
afternoon
Protein Concentration (µg/ml)
30
20
10
0
young, afternoon
old, afternoon
-6
-5
-4
-3
-2
-1
peak
1
2
3
4
5
UC-SEC Fractions

Fig. S1

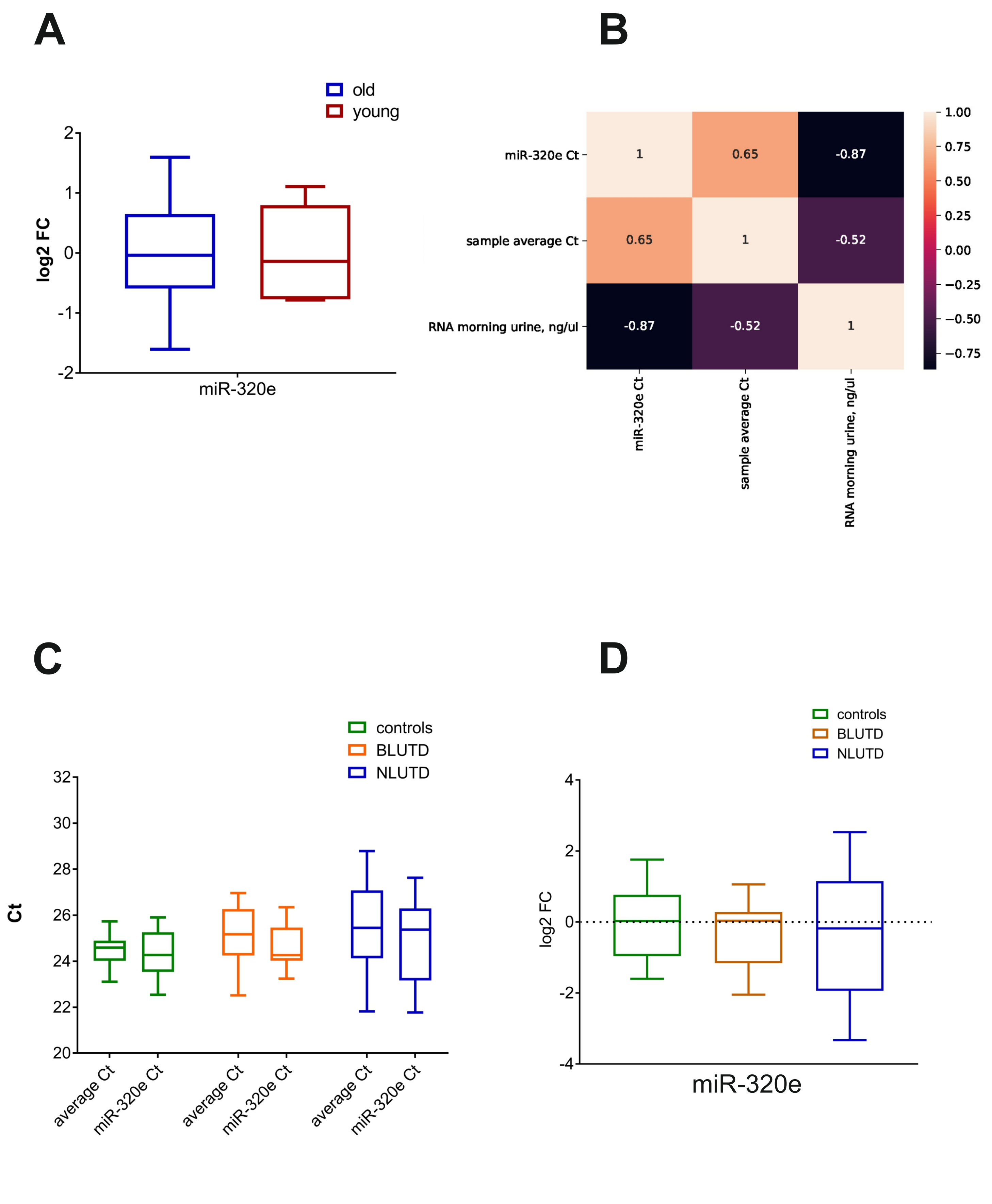
A
old
young
log2 FC
2
1
0
-1
-2
miR-320e
B
miR-320e Ct
sample average Ct
RNA morning urine, ng/ul
1
0.65
-0.87
0.65
1
-0.52
-0.87
-0.52
1
1.00
0.75
0.50
0.25
0.00
−0.25
−0.50
−0.75
C
controls
BLUTD
NLUTD
Ct
32
30
28
26
24
22
20
average Ct
miR-320e Ct
average Ct
miR-320e Ct
average Ct
miR-320e Ct
D
controls
BLUTD
NLUTD
log2 FC
4
2
0
-2
-4
miR-320e

Fig. S2

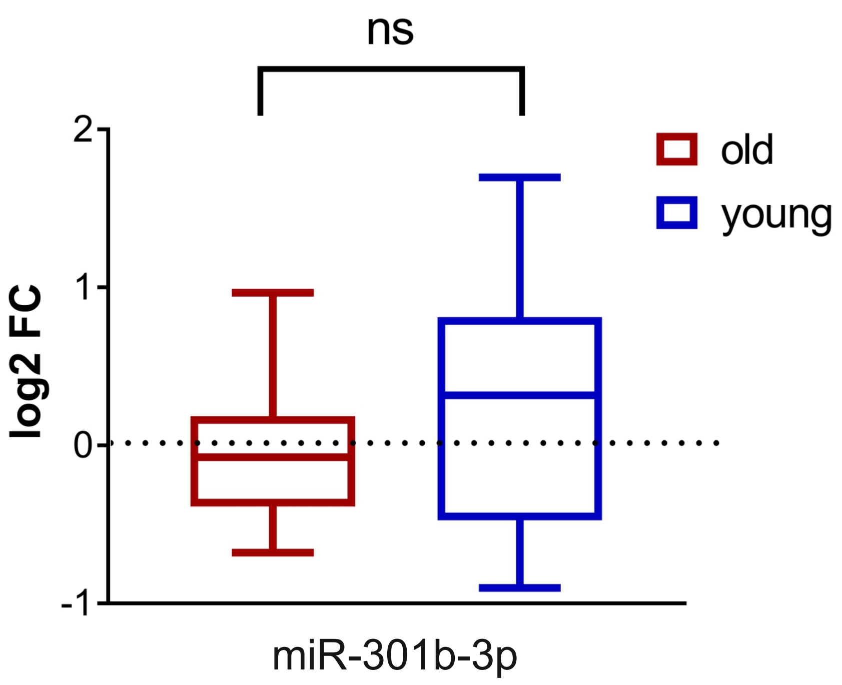


Fig. S3

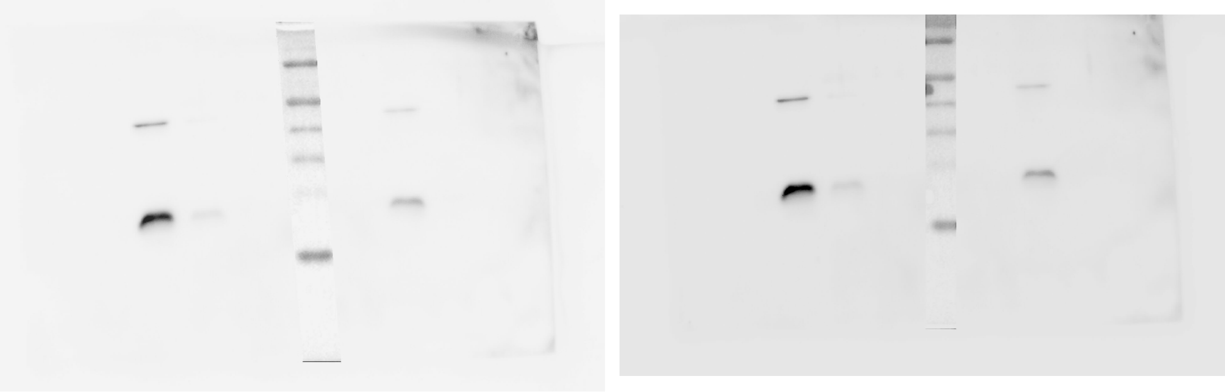

young, subject 6

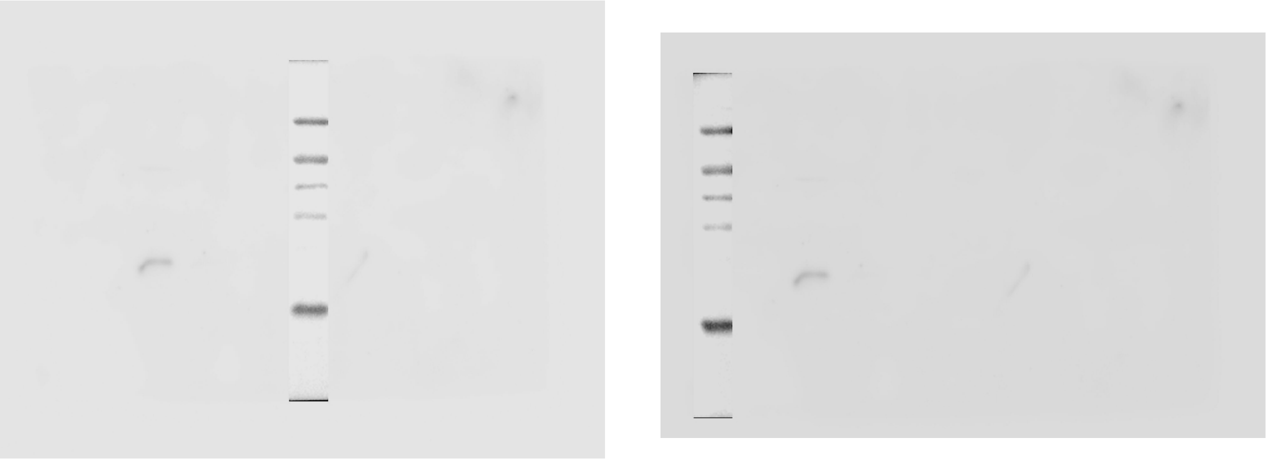

old, subject 10

Fig. S4
